# Computational modelling of acetylcholine deficiency-mediated dysfunction in the basal ganglia-thalamocortical network related to Parkinson’s disease dementia

**DOI:** 10.1371/journal.pone.0361725

**Published:** 2026-11-04

**Authors:** Hao Yang

**Affiliations:** School of Mathematics and Computer Engineering, Ordos Institute of Technology, Ordos, China; Georgia State University, UNITED STATES OF AMERICA

## Abstract

Parkinson’s disease dementia (PDD) is a significant form of dementia. Its pathophysiology involves not only the dopaminergic system impairment but also the degeneration of cholinergic neurons in the pedunculopontine nucleus (PPN), which is considered a potential contributor to the cognitive decline in PDD. Therefore, this study develops a computational model of the PPN-modulated basal ganglia-thalamic-cortical network to investigate how acetylcholine deficiency affects cortical oscillations. Based on anatomical evidence, acetylcholine deficiency is modeled by modulating synaptic connections from the PPN to the thalamus and basal ganglia output nuclei. Power spectrum analysis shows that acetylcholine deficiency, whether in the PPN-thalamic nucleus pathway or in the PPN-basal ganglia output nucleus pathway, leads to a decrease in cortical power ratio between the high-frequency band and low-frequency band. These results qualitatively reproduce the characteristics of electrophysiological measurements recorded in PDD. Further dynamical analysis suggests that this reduction may be associated with weakened oscillatory activity, reflected by a shift in neuronal firing patterns from limit cycle to point attractor. Moreover, simulations of combined acetylcholine and dopamine deficiencies reveal that both neurotransmitters can reduce the cortical power ratio, with a synergistic interaction rather than a simple additive effect. These findings provide a systems-level perspective on how ACh deficiency may contribute to cortical dysfunction in PDD and may offer a potential direction for future therapeutic research.

## 1. Introduction

Parkinson’s disease dementia (PDD) is a secondary neurological disorder of Parkinson’s disease (PD) [1,2]. Its clinical manifestations are primarily marked by a significant progression of cognitive impairment, which not only severely impairs patients’ quality of life but also imposes a substantial burden on public health systems [2,3]. Consequently, understanding the onset characteristics of dementia in PD and elucidating its underlying pathological mechanisms have become critical scientific priorities.

In PDD, cognitive decline is closely accompanied with abnormalities in cerebral neural electrical activity. Numerous studies have demonstrated that patients with PDD exhibit significant alterations in electroencephalogram (EEG). Caviness et al. reported that in comparison to PD patients without dementia, those with PDD show reduced activity in the high-frequency bands (alpha, beta) and increased activity in the low-frequency bands (delta, theta) within cortical regions [4,5]. Morita et al. further reported that the ratio of high-frequency to low-frequency band power is positively correlated with scores on the Mini-Mental State Examination (MMSE) [6]. A lower ratio is associated with decreased MMSE scores, indicating more severe cognitive impairment. In addition, the study revealed that, compared to healthy individuals, PD patients without dementia also exhibit reduced neural activity in the high-frequency band, accompanied by increased activity in the low-frequency band [7,8]. Although these EEG alterations can aid in diagnosing PDD, traditional EEG spectral analysis primarily characterizes the “static” power variations of neural rhythms, which are insufficient for fully characterizing the “dynamic” functional impairments inherent in the brain as a complex dynamical system. To gain a more comprehensive understanding of the neural mechanisms underlying cerebral activity changes in PDD, researchers have increasingly turned to dynamical analysis.

By reducing thousands of interacting neurons to a few population variables, the mean-field neural model can efficiently simulate the neural activity of large-scale networks [9,10]. Given that the basal ganglia represents the core lesion area of PD, its dynamic characteristics naturally become the main focus in PD and PDD research. In 2002, Terman et al. developed an excitatory-inhibitory network model to simulate the interaction between the subthalamic nucleus (STN) and the external globus pallidus (GPe), successfully replicating the pathological beta oscillations observed in patients with PD [11]. Subsequently, Rubin and Terman (2004) extended this model by incorporating the internus globus pallidus (GPi) and thalamic nuclei, demonstrating that high-frequency stimulation of STN significantly enhances thalamic information transmission capacity [12]. In 2006, Leblois et al. proposed a modeling framework based on competitive interactions between the direct and hyperdirect pathways, showing that imbalanced feedback regulation between these two pathways can lead to abnormal oscillations within the network [13]. Furthermore, in 2009, Van Albada and Robinson developed the basal ganglia-thalamocortical circuit (BGTC) model, which demonstrates that dopamine deficiency in PD disrupts the excitatory-inhibitory balance within the cortex-basal ganglia pathway, resulting in altered dynamical discharge patterns across the entire network [14]. However, these models primarily address the neural mechanisms underlying motor dysfunction in PD and do not systematically account for the associated cognitive impairments. To address this limitation, and based on the functional anatomical distinctions between motor and associative circuits in PD, we proposed a parallel circuit model for PDD in 2024, offering a novel theoretical framework for understanding the neurobiological basis of cognitive decline in this condition [15]. In 2025, Ma et al. further explored the impact of VTA dopamine deficiency on neural activity in the basal ganglia-thalamus-cortical network [16].

However, all of the above models have primarily focused on the pathological mechanisms underlying dopaminergic system dysfunction, and none have incorporated cholinergic inputs from the pedunculopontine nucleus (PPN). Recent physiological evidence suggested that cholinergic system dysfunction may also contribute to the onset and progression of PDD. As a secondary cognitive impairment arising during the course of PD, the neuropathology of PDD extended beyond dopaminergic system imbalance and included substantial degeneration of cholinergic neurons [17–19]. The loss of cholinergic neurons was widely recognized as a critical factor underlying cognitive deficits in PD patients [17,18]. Notably, Zweig et al. demonstrated in PD patients a reduction of approximately 40% in the average number of cholinergic neurons within PPN [20]. And this neuronal loss exhibited a significant correlation with the extent of neuronal degeneration in the substantia nigra pars compacta (SNc) [20]. Therefore, a comprehensive investigation into the molecular mechanisms of cholinergic neuron degeneration in the PPN, as well as the synergistic effects of combined acetylcholine (ACh) and dopamine deficits on cognitive function in PDD, may provide useful theoretical insights for a better understanding of PDD pathogenesis.

Accordingly, in this study, we incorporate the PPN (a cholinergic nucleus) into a basal ganglia-thalamus-cortex model (with associative and motor circuits mediated by the caudate nucleus and putamen, respectively; [15]) to explore the effects of ACh deficiency and the combined deficiency of ACh and dopamine on cortical activity. Unlike previous parallel circuit models, this extension enables simulation of PPN degeneration and ACh loss effects on cortical oscillations. The structure of this work is organized as follows: The “Model and methods” section systematically presents the computational framework, dynamical equations, and research methodology; the “Main results” section elucidates the effects of ACh deficiency in motor and associative circuits and its combined deficiency with dopamine on cortical neural activity patterns; the “Discussion and conclusion” section summarizes the key findings, discusses their implications, and outlines the current model’s limitations along with potential directions for future development.

## 2. Model and methods

### 2.1 The kinetic model

As illustrated in Fig 1, based on the existing parallel circuit model [15], this work constructs an enhanced basal ganglia-thalamus-cortex model by incorporating PPN. The model comprises two primary parallel circuits: the motor circuit, which involves the supplementary motor area (including pyramidal neuron a and interneuron b), putamen, GPi, and the ventrolateral thalamic nucleus (VL); and the associative circuit, which includes the prefrontal cortex (including pyramidal neuron e and interneuron i), the caudate nucleus, the substantia nigra pars reticulata (SNr), and the ventral anterior nucleus (VAMC). The synaptic connections between these nuclei, as illustrated in Fig 1, are exclusively glutamate (Glu) or γ-aminobutyric acid (GABA), which is consistent with findings reported in the existing literature [15].

**Fig 1:**
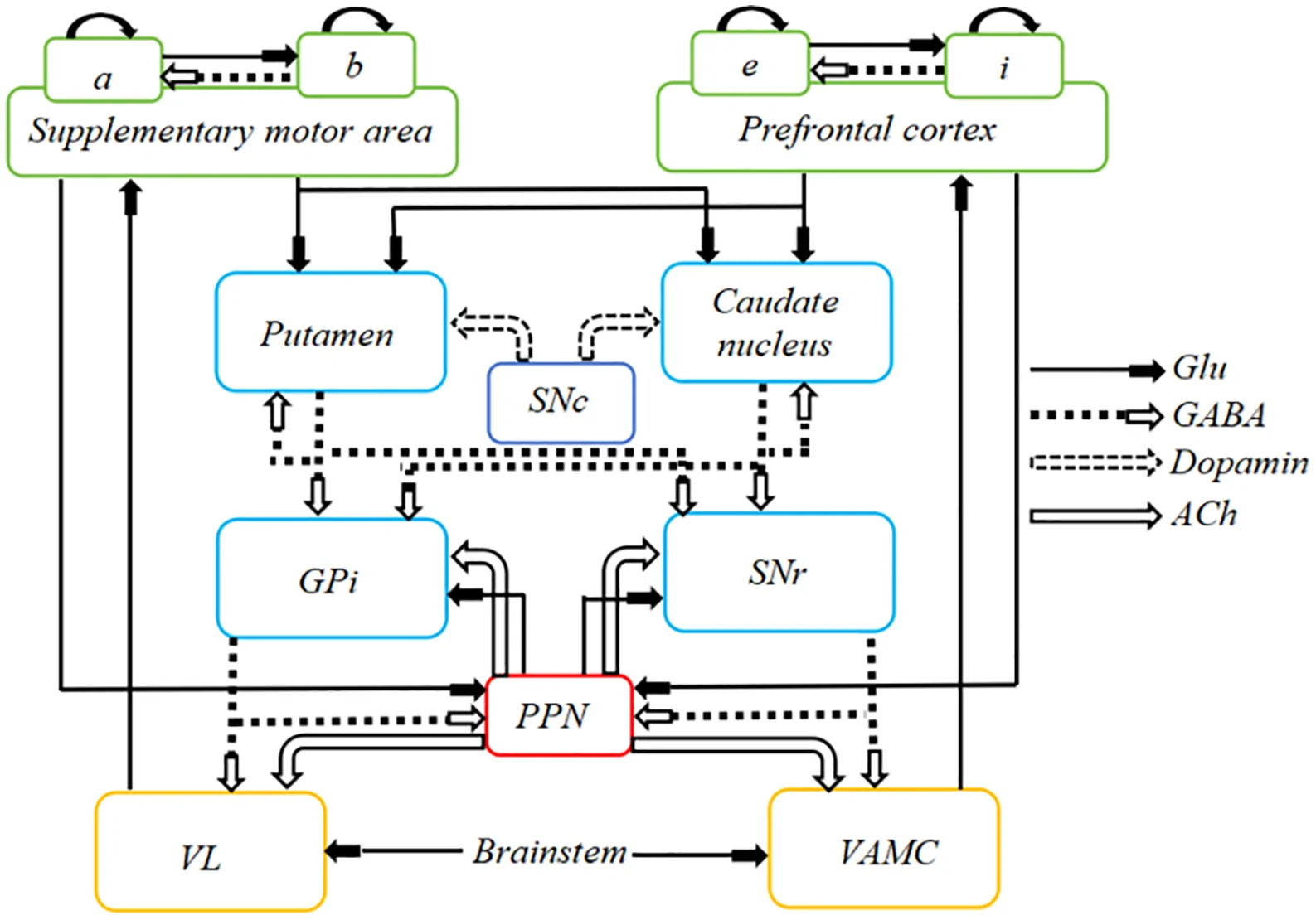
Schematic diagram of the basal ganglia-thalamus-cortex parallel circuit controlled by PPN. The model includes two parallel circuits: the motor circuit, which involves the pyramidal neuron a and interneuron b of supplementary motor area, putamen, GPi, and VL; and the associative circuit, which includes the pyramidal neuron e and interneuron i of the prefrontal cortex, the caudate nucleus, SNr, and VAMC.

The integration of PPN into the model is based on the following physiological evidence: Firstly, considering the cholinergic projection of PPN to the thalamus [21,22], the model realizes the synaptic connection from PPN to VL and VAMC with ACh as neurotransmitter; Secondly, in view of the glutamatergic and cholinergic effects of PPN on the GPi and SNr [23–25], the model incorporates the corresponding glutamate and ACh synaptic connections from PPN to GPi and SNr; Finally, the model contains the downward glutamatergic projection from cortical pyramidal neurons (a and e) to PPN, which is consistent with the experimental findings supporting the existence of the cortical feedback loop [26,27].

The mean-field model treats neurons of the same type as a population and uses population-level dynamical equations to describe the conversion between mean membrane potential and mean firing rate [9,10]. This allows effective simulation of large-scale neuronal activity, and the model has been widely validated against EEG spectra [14]. Therefore, to simulate the model shown in Fig 1, the mean field formulation is used to describe the population dynamics of different brain regions [10,14,15]. The kinetic equations for each population n are as follows:

The mean firing rate $Q_{n}$ of a neural population n is calculated by sigmoid function,


$$\begin{array}{c} Q_{n}(t)=S(V_{n}(t))=\frac{Q_{n}^{max}}{\text{1}+exp(-(V_{n}(t)-\theta_{n})/\sigma )} \end{array}$$ (1)

where $Q_{n}^{max}$ is the maximum achievable firing rate of population n; $\theta_{n}$ is the average threshold potential of population n; and $\sqrt{\text{3}}\sigma /\pi$ is the standard deviation of firing threshold subjected to Gaussian distribution [10,14,15]. $V_{n}(t)$ is the mean cell-body potential of population n, which can be calculated as:


$$\begin{array}{c} D_{\alpha \beta }V_{n}(t)=\sum_{u}A_{nu}\phi_{u}(t-\tau_{nu}) \end{array}$$ (2)

$$\begin{array}{c} D_{\alpha \beta }=\frac{\text{1}}{\alpha \beta }\frac{d^{\text{2}}}{dt^{\text{2}}}+(\frac{\text{1}}{\alpha }+\frac{\text{1}}{\beta })\frac{d}{dt}+\text{1} \end{array}$$ (3)

$A_{nu}$ is the synaptic connectivity parameter from population u to population n. The difference operator $D_{\alpha \beta }$ represents synaptic and dendritic integration of input signals. α and β are the decay and rise rates of cell body potential, respectively. The axonal signal $\phi_{u}(t-\tau_{nu})$ is the input pulse rate from population u.

In a number of previous works [28], neural activity was modeled as spreading along the cortex in a wave-like fashion, based on consistent experimental observations of neural activity upon localized cortical stimulation. Estimates of characteristic axonal ranges and propagation speeds suggest that such waves are significantly damped on the scale of the human cortex. To capture this damped wave propagation, a second-order damped-wave equation is required for the axonal signal $\phi_{u}(t)$,


$$\begin{array}{c} \frac{\text{1}}{\gamma_{u}^{\text{2}}}(\frac{\partial^{\text{2}}}{\partial t^{\text{2}}}+\text{2}\gamma_{u}\frac{\partial }{\partial t}+\gamma_{u}^{\text{2}})\phi_{u}(t)=Q_{u}(t) \end{array}$$ (4)

$\gamma_{u}=\upsilon /r_{u}$ is the damping rate, in which υ is average conduction velocity and $r_{u}$ is characteristic axonal range of population u. $\tau_{nu}$ is the axonal delay from population u to n. In practice, except for cortical pyramidal neurons, the characteristic axonal range $r_{u}$ of other neurons being very short leads to a large damping rate $\gamma_{u}$, which can be considered to tend towards ∞. As $\gamma_{u}\to \infty$, the term $\text{1}/\gamma_{u}^{\text{2}}\to \text{0}$ in the damped-wave equation, and the dynamic is assumed as $\phi_{u}(t)=Q_{u}(t)$. In a word, in this framework, the cortical pyramidal neuron population e and a are described by second-order damped oscillatory equations, while other neuron populations with extremely short axonal ranges are described by $\phi_{u}(t)=Q_{u}(t)$.

For convenience, we use subscripts e, i, a, b, p, c, g, s, l, m, and k to represent the pyramidal neuron and inhibitory interneuron in the prefrontal cortex, the pyramidal neuron and inhibitory interneuron in the supplementary motor area, the putamen nucleus, the caudate nucleus, GPi, SNr, VL, VAMC, and PPN population, respectively. The correspondence between each neural population and its subscript is summarized in Table 1. Please note that since PPN to GPi and SNr have both glutamate and ACh synapses, the glutamate and cholinergic synaptic connections from PPN to GPi are represented by $A_{gk}$ and ${ACh}_{gki}$, respectively. The Glu and cholinergic synaptic connections from PPN to SNr are represented by $A_{sk}$ and ${ACh}_{ski}$, respectively. At the same time, for the convenience of the subsequent research, the cholinergic synaptic connections from PPN to VL and VAMC are respectively represented by ${ACh}_{lke}$ and ${ACh}_{mke}$. In addition, the input pulse rate and the synaptic connectivity parameters from brainstem to population VL and VAMC are abbreviated as $\varphi_{in}$、$A_{lin}$ and $A_{min}$ respectively. The overall model is as follows:

**Table 1:** Correspondence between neural populations and subscript symbols.

Population	Subscript	Population	Subscript
Pyramidal neuron population of supplementary motor area	a	Pyramidal neuron population of prefrontal cortex	e
Interneuron population of supplementary motor area	b	Pyramidal neuron population of prefrontal cortex	i
Putamen	p	Caudate nucleus	c
GPi	g	SNr	s
VL	l	VAMC	m
PPN	k		


$$\begin{array}{cc} 𝐚:\;V_{a}^{\prime \prime }(t)= & \alpha \beta \left[A_{aa}\phi_{a}\left(t-\tau_{aa}\right)+A_{al}\phi_{l}\left(t-\tau_{al}\right)+A_{ab}\phi_{b}\left(t-\tau_{ab}\right)\right]\\ & -(\alpha +\beta )V_{a}^{\prime }(t)-\alpha \beta V_{a}(t)\;Q_{a}(t)=S(Q_{a}^{max}/(\text{1}+exp(-(V_{a}(t)-\theta_{a})/\sigma )))\;\phi_{a}^{\prime \prime }(t)=\gamma_{a}^{\text{2}}Q_{a}(t)-\text{2}\gamma_{a}\phi_{a}^{\prime }(t)-\gamma_{a}^{\text{2}}\phi_{a}(t) \end{array}$$ (5)

$$\begin{array}{cc} b:\;V_{b}^{\prime \prime }(t)= & \alpha \beta [A_{ba}\phi_{a}(t-\tau_{aa})+A_{bb}\phi_{b}(t-\tau_{bb})+A_{bl}\phi_{l}(t-\tau_{bl})]\\ & -(\alpha +\beta )V_{b}^{\prime }(t)-\alpha \beta V_{b}(t)\;\phi_{b}(t)=Q_{b}(t)=S(Q_{b}^{max}/(\text{1}+exp(-(V_{b}(t)-\theta_{b})/\sigma ))) \end{array}$$ (6)

$$\begin{array}{cc} e:\;V_{e}^{\prime \prime }(t)= & \alpha \beta [A_{ee}\phi_{e}(t-\tau_{ee})+A_{em}\phi_{m}(t-em)+A_{ei}\phi_{i}(t-\tau_{ei})]-(\alpha \\ & +\beta )V_{e}^{\prime }(t)-\alpha \beta V_{e}(t)Q_{e}(t)=S(Q_{e}^{max}/(\text{1}+exp(-(V_{e}(t)-\theta_{e})/\sigma )))\;\phi_{e}^{\prime \prime }=\gamma_{e}^{\text{2}}Q_{e}(t)-\text{2}\gamma_{e}\phi_{e}^{\prime }(t)-\gamma_{e}^{\text{2}}\phi_{a}(t) \end{array}$$ (7)

$$\begin{array}{cc} i:\;V_{i}^{\prime \prime }(t)= & \alpha \beta \left[A_{ie}\phi_{e}\left(t-\tau_{ie}\right)+A_{ii}\phi_{i}\left(t-\tau_{ii}\right)+A_{im}\phi_{m}\left(t-\tau_{im}\right)\right]\\ & -(\alpha +\beta )V_{i}^{\prime }(t)-\alpha \beta V_{i}(t)\;\phi_{i}(t)=Q_{i}(t)=S(Q_{i}^{max}/(\text{1}+exp(-(V_{i}(t)-\theta_{i})/\sigma ))) \end{array}$$ (8)

$$\begin{array}{cc} p:\;V_{p}^{\prime \prime }(t)= & \alpha \beta \left[A_{pe}\phi_{e}\left(t-\tau_{pe}\right)+A_{pa}\phi_{a}\left(t-\tau_{pa}\right)+A_{pp}\phi_{p}(t)\right]\\ & -(\alpha +\beta )V_{p}^{\prime }(t)-\alpha \beta V_{p}(t)\;\phi_{p}(t)=Q_{p}(t)=S(Q_{p}^{max}/(\text{1}+exp(-(V_{p}(t)-\theta_{p})/\sigma ))) \end{array}$$ (9)

$$\begin{array}{cc} c:\;V_{c}^{\prime \prime }(t)= & \alpha \beta \left[A_{ce}\phi_{e}\left(t-\tau_{ce}\right)+A_{ca}\phi_{a}\left(t-\tau_{ca}\right)+A_{cc}\phi_{c}(t)\right]\\ & -(\alpha +\beta )V_{c}^{\prime }(t)-\alpha \beta V_{c}(t)\;\phi_{c}(t)=Q_{c}(t)=S(Q_{c}^{max}/(\text{1}+exp(-(V_{c}(t)-\theta_{c})/\sigma ))) \end{array}$$ (10)

$$\begin{array}{cc} s:\;V_{s}^{\prime \prime }(t)= & \alpha \beta \left[A_{sc}\phi_{c}\left(t-\tau_{sc}\right)+A_{sp}\phi_{p}\left(t-\tau_{sp}\right)+A_{sk}\phi_{k}\left(t-\tau_{sk}\right)+{ACh}_{ski}\phi_{k}\left(t-\tau_{sk}\right)\right]\\ & -(\alpha +\beta )V_{s}^{\prime }(t)-\alpha \beta V_{s}(t)\;\phi_{s}(t)=Q_{s}(t)=S(Q_{s}^{max}/(\text{1}+exp(-(V_{s}(t)-\theta_{s})/\sigma ))) \end{array}$$ (11)

$$\begin{array}{cc} g:\;V_{g}^{\prime \prime }(t)= & \alpha \beta [A_{gp}\phi_{p}(t-\tau_{gp})+A_{gc}\phi_{c}(t-\tau_{gc})+A_{gk}\phi_{k}(t-\tau_{gk})+{ACh}_{gki}\phi_{k}(t-\tau_{gk})]\\ & -(\alpha +\beta )V_{g}^{\prime }(t)-\alpha \beta V_{g}(t)\;\phi_{g}(t)=Q_{g}(t)=S(Q_{g}^{max}/(\text{1}+exp(-(V_{g}(t)-\theta_{g})/\sigma ))) \end{array}$$ (12)

$$\begin{array}{cc} m:\;V_{m}^{\prime \prime }(t)= & \alpha \beta \left[A_{ms}\phi_{s}\left(t-\tau_{ms}\right)+{ACh}_{mke}\phi_{k}\left(t-\tau_{mk}\right)+A_{min}\phi_{in}\right]\\ & -(\alpha +\beta )V_{m}^{\prime }(t)-\alpha \beta V_{m}(t)\;\phi_{m}(t)=Q_{m}(t)=S(Q_{m}^{max}/(\text{1}+exp(-(V_{m}(t)-\theta_{m})/\sigma ))) \end{array}$$ (13)

$$\begin{array}{cc} l:\;V_{l}^{\prime \prime }(t)= & \alpha \beta [A_{lg}\phi_{g}(t-\tau_{lg})+{ACh}_{lke}\phi_{k}(t-\tau_{lk})+A_{lin}\phi_{in}]\\ & -(\alpha +\beta )V_{l}^{\prime }(t)-\alpha \beta V_{l}(t)\;\phi_{l}(t)=Q_{l}(t)=S(Q_{l}^{max}/(\text{1}+exp(-(V_{l}(t)-\theta_{l})/\sigma ))) \end{array}$$ (14)

$$\begin{array}{cc} k:\;V_{k}^{\prime \prime }(t)= & \alpha \beta \left[A_{ka}\phi_{a}\left(t-\tau_{ka}\right)+A_{ke}\phi_{e}\left(t-\tau_{ke}\right)\right]\\ & -(\alpha +\beta )V_{k}^{\prime }(t)-\alpha \beta V_{k}(t)\;\phi_{k}(t)=Q_{k}(t)=S(Q_{k}^{max}/(\text{1}+exp(-(V_{k}(t)-\theta_{k})/\sigma ))) \end{array}$$ (15)

All model parameters, except for the four PPN-specific cholinergic parameters (${AC\text{h}}_{gki}$, ${AC\text{h}}_{ski}$, ${AC\text{h}}_{lke}$ and ${AC\text{h}}_{mke}$), are adopted directly from the validated basal ganglia-thalamocortical model in the original literature 15. The values are listed in Tables 2, 3, and 4, and remain fixed throughout all simulations. These parameters are derived from the existing model [14,15,29,32], which was rigorously validated against experimental data. The PPN maximum firing rate and threshold potential are set to ${\text{2}\text{0}\text{0}s}^{-\text{1}}$ and 9mV, respectively [29]. The four cholinergic parameters ${ACh}_{gki}$(from PPN to GPi), ${ACh}_{ski}$(from PPN to SNr), ${ACh}_{lke}$(from PPN to VL) and ${ACh}_{mke}$(from PPN to VAMC) are systematically varied to simulate different degrees of ACh deficiency.

**Table 2:** The values of the maximum firing rate $Q_{n}^{max}$ (unit: $s^{-1}$) and the average threshold potential (unit: mV) for population n.

$Q_{e}^{max}$	$Q_{i}^{max}$	$Q_{a}^{max}$	$Q_{b}^{max}$	$Q_{p}^{max}$	$Q_{c}^{max}$	$Q_{g}^{max}$	$Q_{s}^{max}$	$Q_{m}^{max}$	$Q_{l}^{max}$
300	300	300	300	65	65	250	250	300	300
$\theta_{e}$	$\theta_{i}$	$\theta_{a}$	$\theta_{b}$	$\theta_{p}$	$\theta_{c}$	$\theta_{g}$	$\theta_{s}$	$\theta_{m}$	$\theta_{l}$
14	14	14	14	19	19	10	10	13	13

**Table 3:** The values of the synaptic connection parameter $A_{nu}$ (unit: mVs) and axonal delay (unit: ms) from population u to n.

$A_{ee}$	$A_{ei}$	$A_{em}$	$A_{ie}$	$A_{im}$	$A_{ii}$	$A_{ca}$	$A_{ce}$	$A_{cc}$	$A_{sc}$	$A_{sp}$	$A_{ms}$	$A_{mn}$
1.6	−1.2	0.8	1.6	0.2	−0.2	0.1	0.7	−0.3	−0.1	−0.04	−0.03	0.5
$\tau_{ee}$	$\tau_{ei}$	$\tau_{em}$	$\tau_{ie}$	$\tau_{im}$	$\tau_{ii}$	$\tau_{ca}$	$\tau_{ce}$	$\tau_{cc}$	$\tau_{sc}$	$\tau_{sp}$	$\tau_{ms}$	
0	0	35	0	35	0	2	2	0	1	1	3	
$A_{aa}$	$A_{ab}$	$A_{al}$	$A_{ba}$	$A_{bl}$	$A_{bb}$	$A_{pe}$	$A_{pa}$	$A_{pp}$	$A_{gp}$	$A_{gc}$	$A_{lg}$	$A_{ln}$
1.6	−1.2	0.8	1.6	0.2	−0.2	0.1	0.7	−0.3	−0.1	−0.04	−0.03	0.5
$\tau_{aa}$	$\tau_{ab}$	$\tau_{al}$	$\tau_{ba}$	$\tau_{bl}$	$\tau_{bb}$	$\tau_{pe}$	$\tau_{pa}$	$\tau_{pp}$	$\tau_{gp}$	$\tau_{gc}$	$\tau_{lg}$	
0	0	35	0	35	0	2	2	0	1	1	3	

**Table 4:** Other parameter values.

σ	$\gamma_{e}$	$\gamma_{a}$	α	β
3.3	125$s^{-\text{1}}$	125$s^{-\text{1}}$	160$s^{-\text{1}}$	640$s^{-\text{1}}$

### 2.2 Methods

A large body of studies have demonstrated that ACh plays a dual role in the central nervous system, exerting both excitatory and inhibitory effects depending on the type of receptors [30]. In this study, cholinergic projections from PPN to the thalamus (VL and VAMC) are primarily mediated by muscarinic acetylcholine receptors 1/3 (M1/M3) [21]. As synaptic transmission via these receptors is predominantly excitatory, the cholinergic synaptic connection parameters ${ACh}_{lke}$ and ${ACh}_{mke}$ from PPN to thalamus are assigned positive values [21,30]. Therefore, by reducing the values of ${ACh}_{lke}$ and ${ACh}_{mke}$, the physiological response of the thalamus to the degeneration of PPN cholinergic neurons is simulated. Conversely, cholinergic input from the PPN to the GPi/SNr appears to have an inhibitory effect. Evidence from prior studies indicates that activation of muscarinic acetylcholine receptors (mAChRs) in the globus pallidus suppresses neuronal firing activity [31]. Accordingly, the cholinergic synaptic connection parameters ${ACh}_{gki}$ and ${ACh}_{ski}$ from PPN to GPi/SNr are set to negative values. And the physiological condition of ACh deficiency from PPN to GPi/SNr is simulated by decreasing the absolute values of ${ACh}_{gki}$ and ${ACh}_{ski}$ (${|ACh}_{gki}|$ and ${|ACh}_{ski}|$).

In the following, the second-order differential equations governing the model in Fig. 1 are numerically solved by the explicit Euler method. The simulation is conducted in the MATLAB 2019a over a total duration of 10s, with a fixed time step of ${\text{1}\text{0}}^{-\text{3}}s$ (sampling frequency 1000 Hz). The simulation is initiated with all state variables set to zero. Based on the time series data generated by the model, the power spectral density (PSD) is subsequently computed using Pwelch function in MATLAB with a Hamming window and the following parameters: window length of 1000 samples, overlap of 500 samples (50% overlap), FFT length of 1024 points, and a bandpass filter between 0.1 Hz and 40 Hz. Then, we compute the power ratio by first integrating PSD of the variables $\phi_{a}$ and $\phi_{e}$ over the high-frequency band (8–35 Hz) and over the low-frequency band (1–7.5 Hz), and then dividing the integrated high-frequency power by the integrated low-frequency power, which are closely related to scalp EEG signals in the supplementary motor area and prefrontal cortex [32].

## 3. Main results

In this section, we investigate the effects of ACh deficiency on the supplementary motor area and prefrontal cortex neural activity within the model. Firstly, ACh deficiency in the motor circuit is simulated by reducing ${ACh}_{lke}$ (the cholinergic synaptic connection strength from PPN to VL) and ${|ACh}_{gki}|$ (the cholinergic synaptic connection strength from PPN to GPi), thereby investigating how ACh deficiency in the motor circuit modulates cortical neural activity. Subsequently, ACh deficiency in the associative circuit is modeled by decreasing ${ACh}_{mke}$ (the cholinergic synaptic connection strength from PPN to VAMC) and $\left|{ACh}_{ski}\right|$ (the cholinergic synaptic connection strength from PPN to SNr), allowing for an examination of its influence on cortical neural dynamics. Finally, according to the classical view (the absence of dopamine can enhance the inhibitory output from the striatum to the thalamus) [15,33], dopamine deficiency is simulated by enhancing parameters ${|A}_{lg}|$ (the GABA synaptic connection strength from GPi to VL) and ${|A}_{ms}|$(the GABA synaptic connection strength from SNr to VAMC), enabling an exploration of the combined impact of ACh and dopamine dual neurotransmitter deficiencies on cortical neural activity.

### 3.1 The impact of ACh deficiency in the motor circuit on cortical activity

This subsection investigates the impact of ACh deficiency in the motor circuit on neural activity within the cortical region. Fig 2a and 2b present two-dimensional projections of the power ratios of $\phi_{a}$ and $\phi_{e}$ as the change of parameters ${ACh}_{lke}$ and ${|ACh}_{gki}|$, respectively. Overall, both power ratios decrease as the values of ${ACh}_{lke}$ and ${|ACh}_{gki}|$ diminish; however, the reduction in $\phi_{a}$ is substantially more pronounced (declining from approximately 7000 to nearly 0) whereas the change in $\phi_{e}$ is comparatively modest. The trends of these powers are consistent with the EEG slowing observed in PD patients [7,8]. To observe these changes more intuitively, we conduct simulations under variations of a single parameter while holding the other constant. As illustrated in Fig 2c and 2e, when ${ACh}_{lke}$ (or ${|ACh}_{gki}|$) is fixed, the $\phi_{a}$ power ratio exhibits a marked decline with decreasing values of ${|ACh}_{gki}|$ (or ${ACh}_{lke}$), indicating that ACh depletion in the motor circuit induces significant disruptions in the supplementary motor area’s activity, regardless of whether the effect is mediated through GPi or VL. In contrast, the behavior of the $\phi_{e}$ power ratio is more nuanced (Fig 2d and 2f): it decreases with reduced ${|ACh}_{gki}|$ at ${ACh}_{lke}=\text{0}.\text{2}$, but shows a slight increase at ${ACh}_{lke}=\text{0}.\text{1}$ and ${ACh}_{lke}=\text{0}$. These indicate that the initial deficiency of ACh in the motor circuit leads to a reduction in prefrontal cortex neural activities. However, under severe deficiency conditions, a slight increase in prefrontal cortex neural activity may occur.

**Fig 2:**
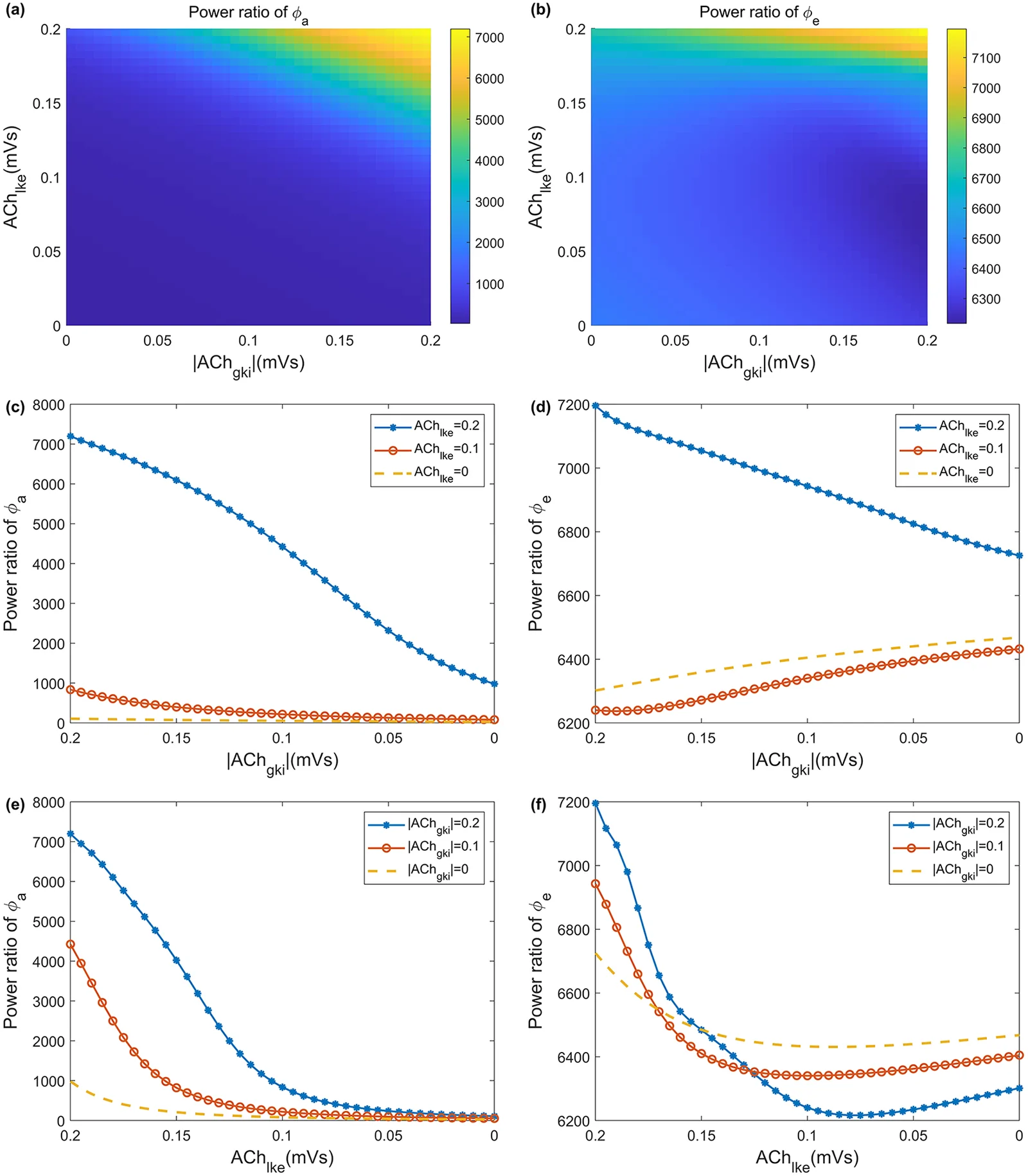
The projection diagrams illustrating the power ratio of $\phi_{a}$ (a) and $\phi_{e}$ (b) with ${ACh}_{lke}$ and ${|ACh}_{gki}|$. The variation of the power ratio of $\phi_{a}$ (c) and $\phi_{e}$ (d) with the decrease of ${|ACh}_{gki}|$ under fixed ${ACh}_{lke}$. The variation of the power ratio of $\phi_{a}$ (e) and $\phi_{e}$ (f) with the decrease of ${ACh}_{lke}$ under fixed ${|ACh}_{gki}|$.

Fig 3 displays the PSD curves of $\phi_{a}$ and $\phi_{e}$ under various parameter combinations. As shown in the PSD of $\phi_{a}$ (Fig 3a), as the values of ${ACh}_{lke}$ and $\left|{ACh}_{gki}\right|$ decrease, high-frequency band power is progressively attenuated relative to low-frequency components. This attenuation is most prominent when ${ACh}_{lke}=\text{0}$ and ${|ACh}_{gki}|=\text{0}$, where high-frequency power is nearly abolished. Comparative analysis indicates that the reduction in ${ACh}_{lke}$ exerts a stronger suppressive effect on the $\phi_{a}$ power ratio than does the reduction in ${|ACh}_{gki}|$. For $\phi_{e}$ (Fig 3b), parameter variations do not markedly alter the overall spectral shape of the PSD; and the peaks in the high-frequency bands are slightly diminished across other conditions, resulting in an overall reduction in the power ratio.

**Fig 3:**
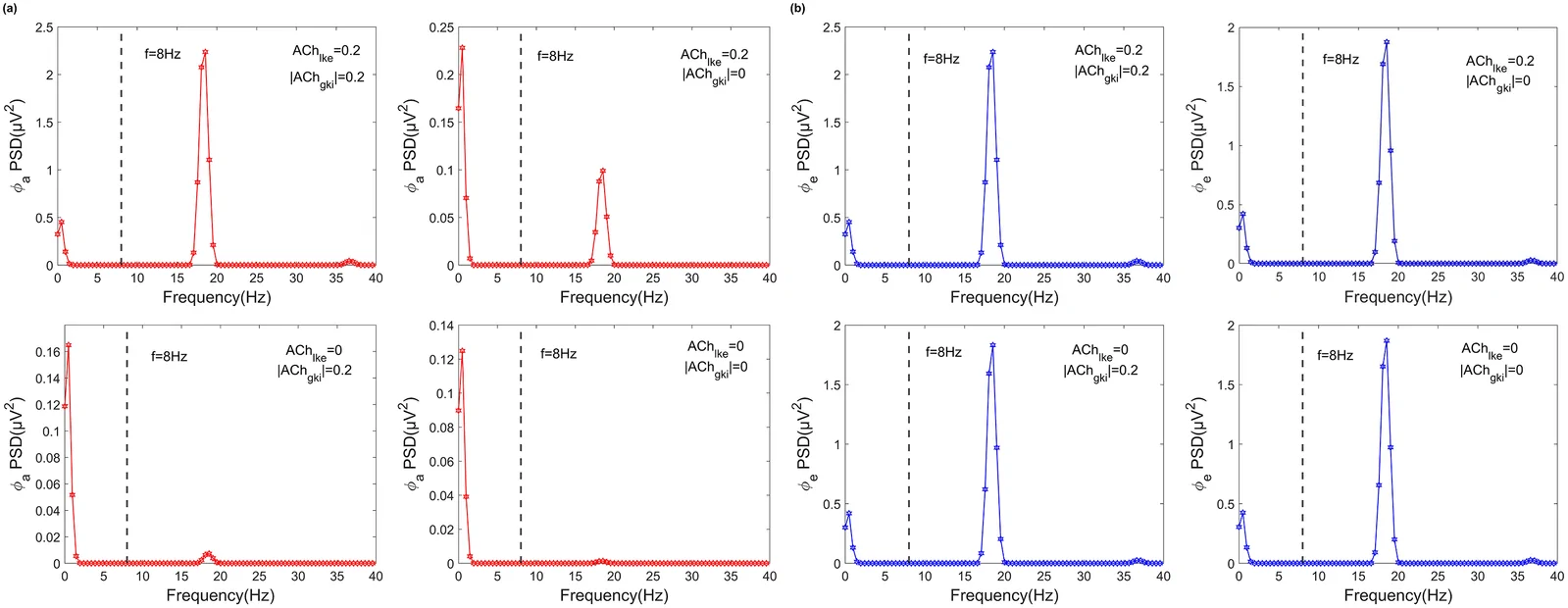
Power spectrum density diagrams of $\phi_{a}$ (a) and $\phi_{e}$ (b) for different combinations of ${ACh}_{lke}$ and ${|ACh}_{gki}|$. From left to right, ${|ACh}_{gki}|$ decreases progressively (from 0.2 to 0), and from top to bottom, ${ACh}_{lke}$ decreases progressively (from 0.2 to 0), representing increasing severity of ACh deficiency in the motor circuit.

We further examine the dynamical behavior of the mean membrane potentials $V_{a}$ and $V_{e}$ in the cortical region as the values of ${ACh}_{lke}$ and ${|ACh}_{gki}|$ decreased. As illustrated in Fig 4a, the oscillation amplitude of $V_{a}$ progressively diminishes with the reduction in values of ${ACh}_{lke}$ and ${|ACh}_{gki}|$, eventually approaching zero. This trend aligns closely with the decline in the power ratio of $\phi_{a}$. The corresponding phase portrait (Fig 4c) clearly illustrates this evolution: as the absolute values of the parameters decrease, the system’s limit cycle gradually contracts, ultimately collapsing into a stable fixed point when both ${ACh}_{lke}$ and ${|ACh}_{gki}|$ reach zero.

**Fig 4:**
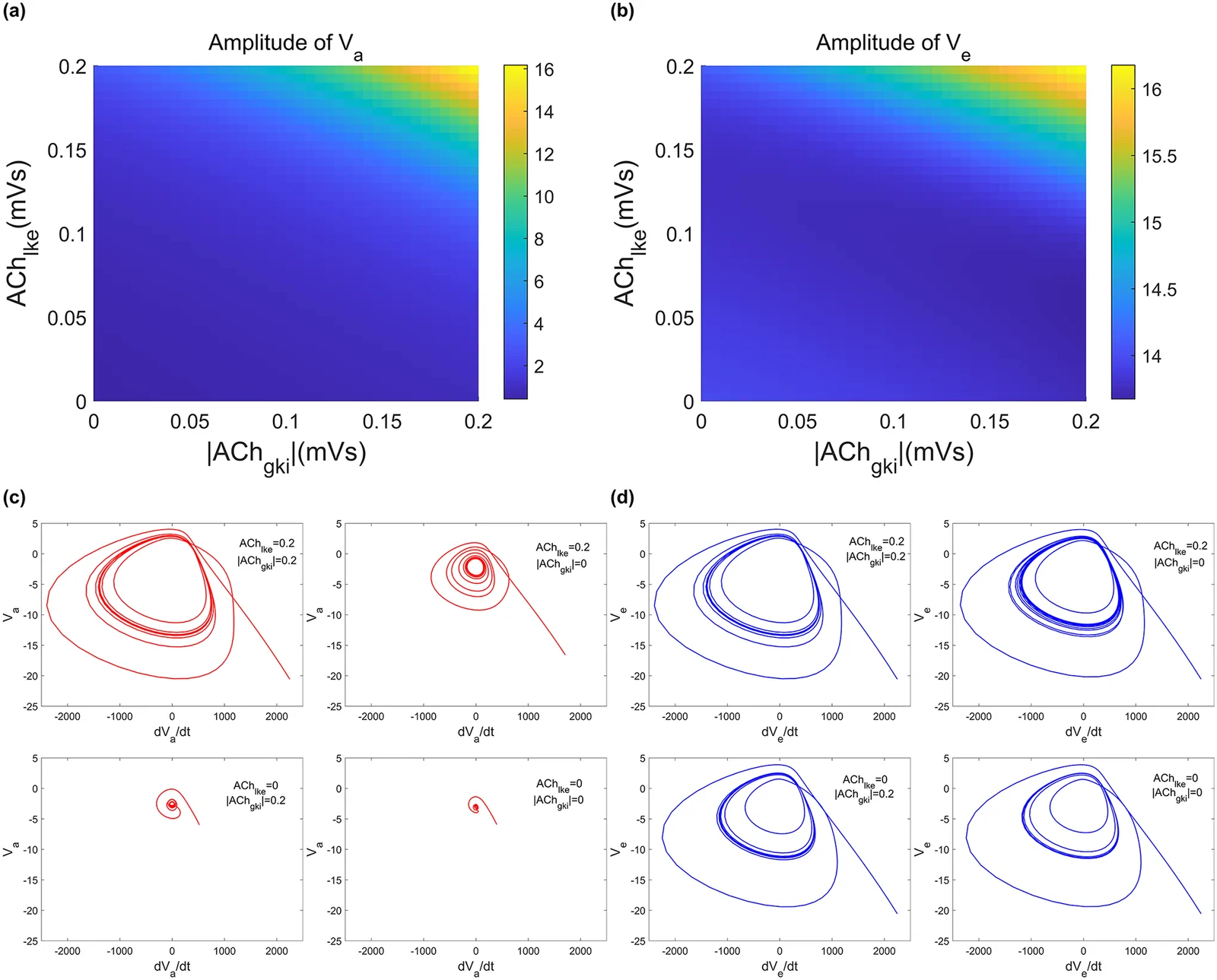
The projection diagrams of the amplitudes of $V_{a}$ (a) and $V_{e}$ (b) varying with ${ACh}_{lke}$ and ${|ACh}_{gki}|$. The phase planar graph of $V_{a}$ and its derivatives (c), the phase planar graph of $V_{e}$ and its derivatives (d) with respect to ${ACh}_{lke}$ and ${|ACh}_{gki}|$. From left to right, ${|ACh}_{gki}|$ decreases progressively (from 0.2 to 0), and from top to bottom, ${ACh}_{lke}$ decreases progressively (from 0.2 to 0), representing increasing severity of ACh deficiency in the motor circuit.

Similarly, as shown in Fig 4b, the amplitude of $V_{e}$ generally decreases as the extent of parameter deviation increases, which is consistent with the overall trend observed in the power ratio of $\phi_{e}$. However, a slight increase in the amplitude of $V_{e}$ is observed when ${ACh}_{lke}<\text{0}.\text{1}$ and the value of ${|ACh}_{gki}|$ further decreases. As shown in the phase plane diagram (Fig 4d), the limit cycle of $V_{e}$ undergoes only minimal contraction across varying parameter conditions, suggesting that its dynamical state remains relatively stable and is less sensitive to disruptions in ACh signaling.

To more intuitively illustrate the changes in mean membrane potentials $V_{a}$($V_{e}$) and related pulse rates $\phi_{a}$($\phi_{e}$), we present the time series of $V_{a}$(a) and $V_{e}$(b) for different ${ACh}_{lke}$ and ${|ACh}_{gki}|$ in Fig 5. As shown in the Fig 5a, with the progressive reduction of ${ACh}_{lke}$ and ${|ACh}_{gki}|$, the amplitude of membrane potential oscillations in $V_{a}$ gradually decreases. When ${ACh}_{lke}$ and ${|ACh}_{gki}|$ are both reduced to zero, $V_{a}$ exhibits almost no oscillatory activity, indicating a near-complete collapse of rhythmic firing. This loss of oscillatory amplitude underlies the observed decrease in the power spectral density of $\phi_{a}$, leading to a progressive decline in the power ratio. In contrast, under the same conditions, the oscillatory state of $V_{e}$ remains largely unchanged, showing no significant reduction in amplitude.

**Fig 5:**
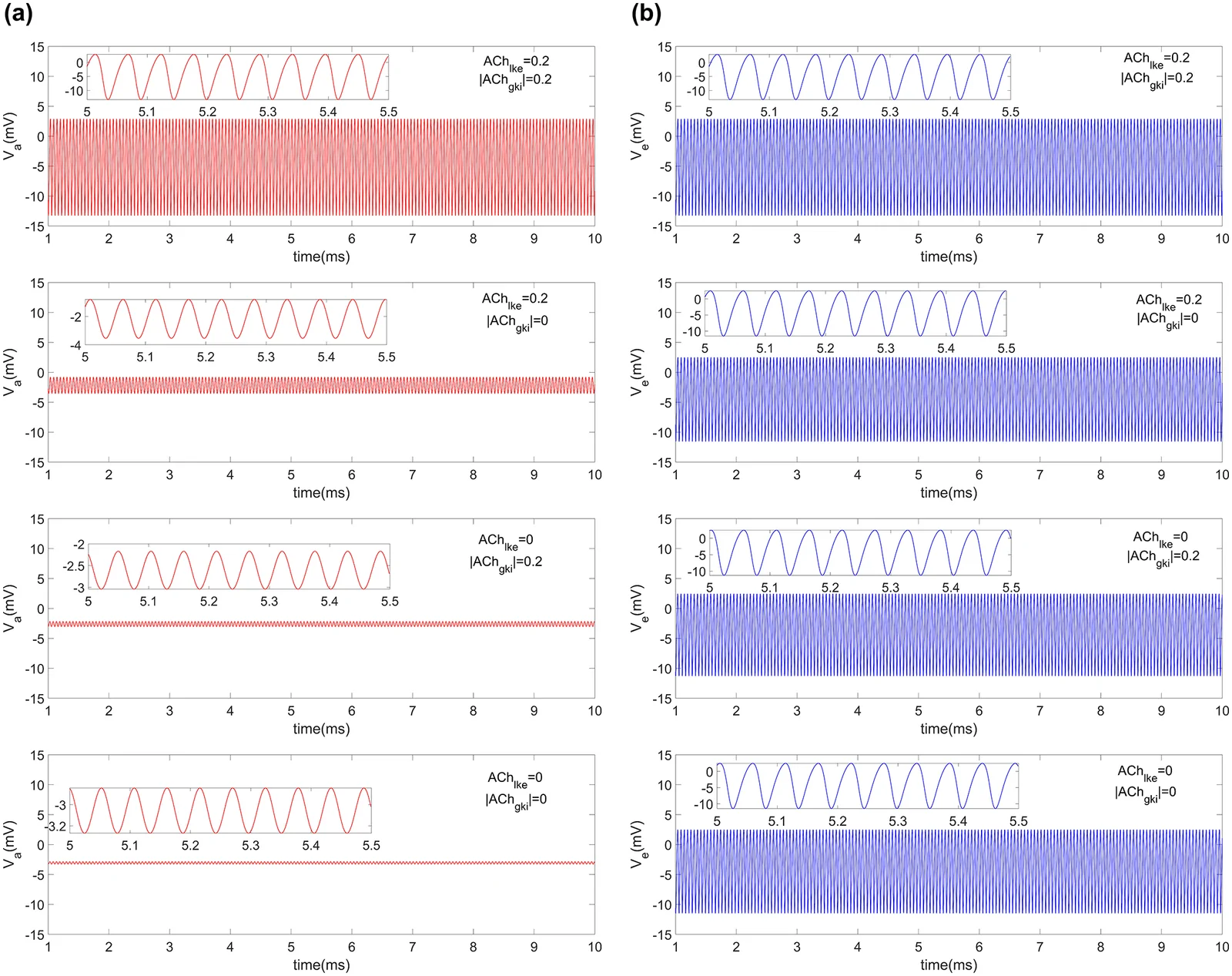
The time series of $V_{a}$ (panel a) and $V_{e}$ (panel b) for different ${ACh}_{lke}$ and ${|ACh}_{gki}|$. ${|ACh}_{gki}|$ decreases progressively (from 0.2 to 0) and ${ACh}_{lke}$ decreases progressively (from 0.2 to 0), representing increasing severity of ACh deficiency in the motor circuit.

### 3.2 The impact of ACh deficiency in the associative circuit on cortical activity

In this subsection, the impact of ACh deficiency within the associative circuit on neural oscillatory activity is investigated through modulation of the parameters ${ACh}_{mke}$ and ${|ACh}_{ski}|$. As illustrated in Fig 6a, the $\phi_{a}$ power ratio exhibits a general downward trend as the values of ${ACh}_{mke}$ and ${|ACh}_{ski}|$ decrease, which aligns with EEG alterations observed in PD [7,8]. Fig 6b indicates a more pronounced reduction in the $\phi_{e}$ power ratio, consistent with clinical findings associated with dementia in PDD [4–6]. To examine the individual contributions of each parameter, simulations are performed with one parameter held constant. As shown in Fig 6c and 6d, when ${ACh}_{mke}$ is fixed, the $\phi_{e}$ power ratio progressively decreases with decreasing ${|ACh}_{ski}|$; however, the $\phi_{a}$ power ratio decreases only when ${ACh}_{mke}=\text{0}.\text{2}$ and shows a slight increase at ${ACh}_{mke}=\text{0}.\text{1}$ and ${ACh}_{mke}=\text{0}$. Conversely, when ${|ACh}_{ski}|$ is held constant, both the $\phi_{a}$ and $\phi_{e}$ power ratios generally decline with decreasing ${ACh}_{mke}$, as depicted in Fig 6e and 6f.

**Fig 6:**
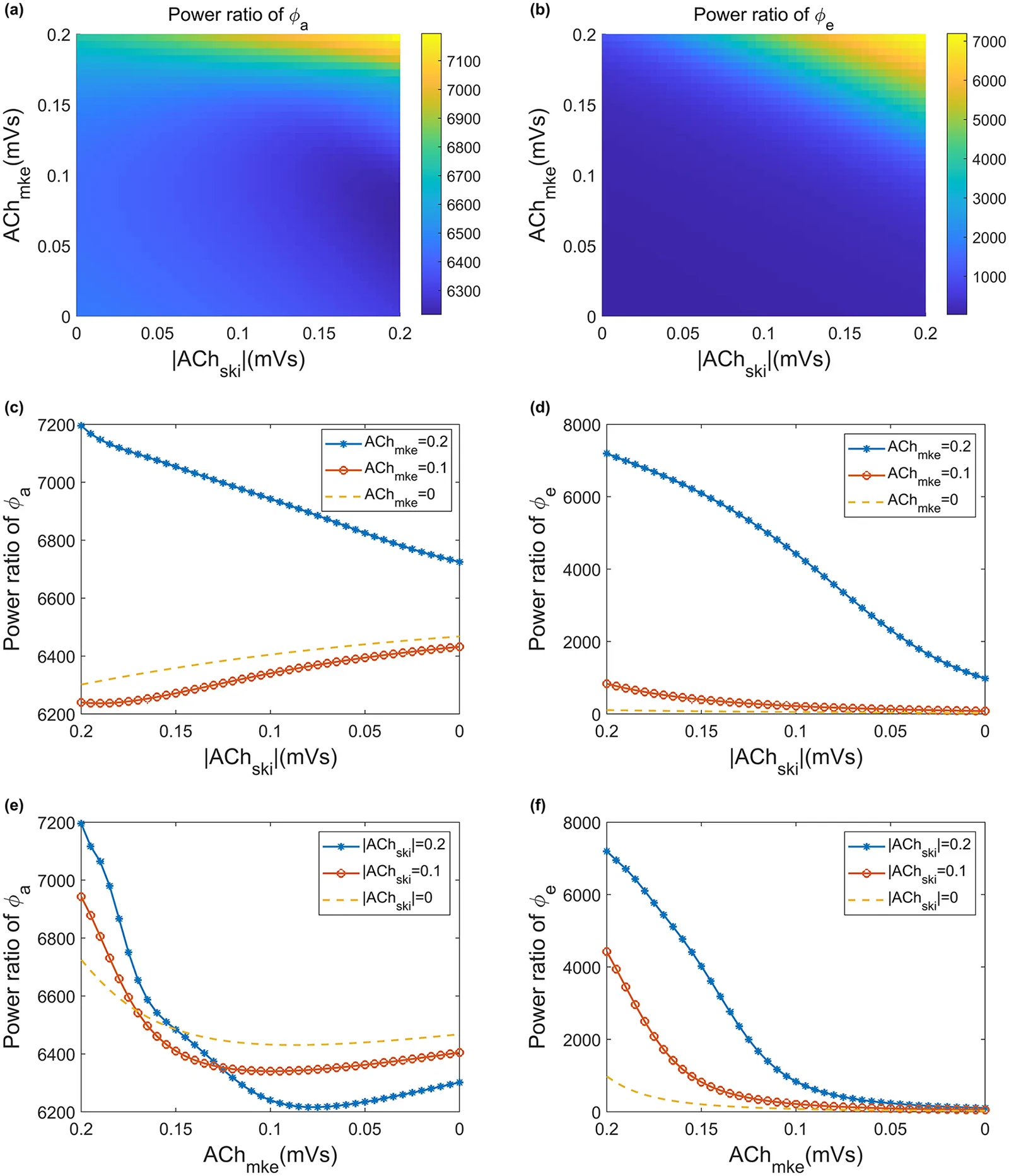
The projection diagrams illustrating the power ratio of $\phi_{a}$ (a) and $\phi_{e}$ (b) with ${ACh}_{mke}$ and ${|ACh}_{ski}|$. The variation of the power ratio of $\phi_{a}$ (c) and $\phi_{e}$ (d) with the decrease of ${|ACh}_{ski}|$ under fixed ${ACh}_{mke}$. The variation of the power ratio of $\phi_{a}$ (e) and $\phi_{e}$ (f) with the decrease of ${ACh}_{mke}$ under fixed ${|ACh}_{ski}|$.

Further analysis reveals that the PSD curve of $\phi_{a}$ remains largely stable across varying parameter conditions, with only a minor reduction in peak amplitude (Fig 7a). In contrast, the PSD of $\phi_{e}$ demonstrates marked changes: as the values of ${ACh}_{mke}$ and ${|ACh}_{ski}|$ decrease, high-frequency band power diminishes significantly relative to the low-frequency band (Fig 7b).

**Fig 7:**
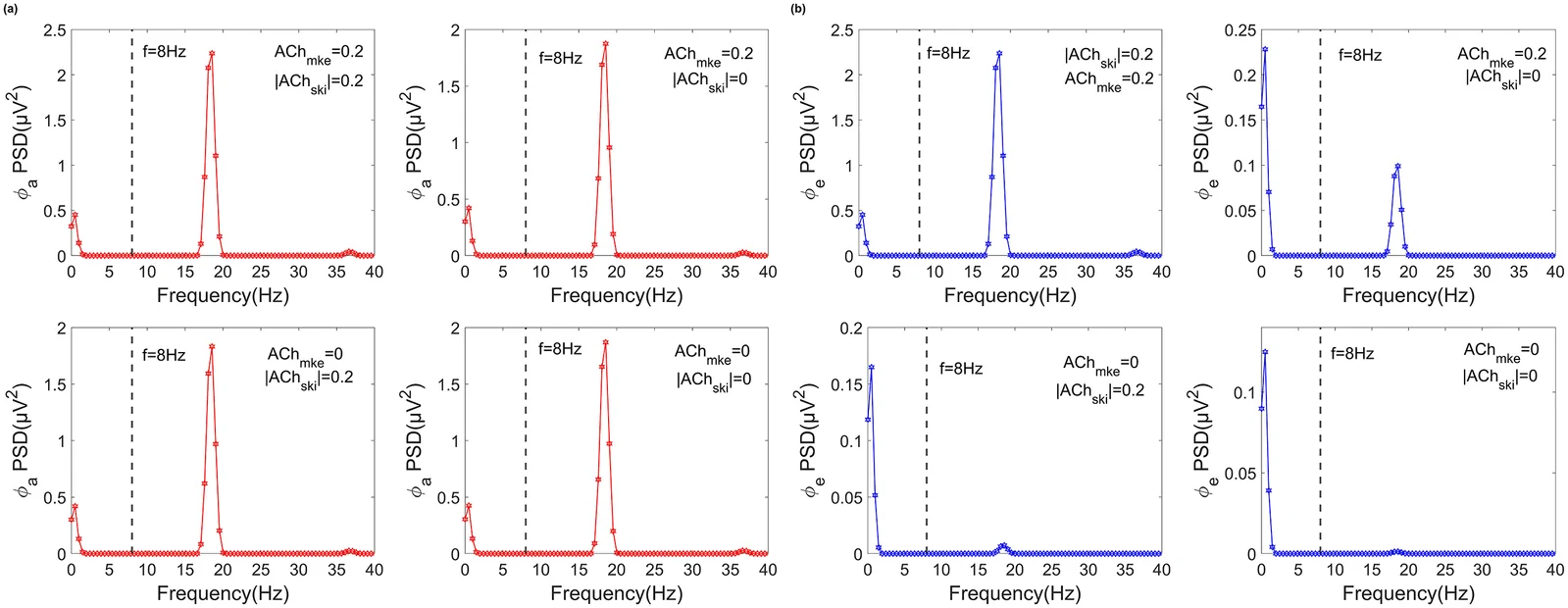
Power spectrum density diagrams of $\phi_{a}$ (a) and $\phi_{e}$ (b) for different combinations of ${ACh}_{mke}$ and ${|ACh}_{ski}$. From left to right, ${|ACh}_{ski}|$ decreases progressively (from 0.2 to 0), and from top to bottom, ${ACh}_{mke}$ decreases progressively (from 0.2 to 0), representing increasing severity of ACh deficiency in the associative circuit.

As illustrated in Fig 8a and 8b, the amplitude variation of the mean membrane potential $V_{a}$ aligns with the overall trend of the $\phi_{a}$ power ratio, exhibiting a reduction as the values of ${ACh}_{mke}$ and ${|ACh}_{ski}|$ decrease. However, when ${ACh}_{mke}<\text{1}$, a slight rebound in the amplitude of $V_{a}$ is observed with further decreases in ${|ACh}_{ski}|$. In contrast, the amplitude of $V_{e}$ progressively diminishes as both parameters are reduced. Corresponding phase plane analysis highlights distinct differences in their dynamical behaviors (Fig 8c and 8d). The phase portraits of $V_{a}$ and its derivative retain essentially the same shape across different parameter combinations, indicating a stable dynamical mode. In comparison, the phase trajectory of $V_{e}$ undergoes significant changes: its limit cycle gradually contracts with decreasing values of ${ACh}_{mke}$ and ${|ACh}_{ski}|$, ultimately collapsing into a fixed point when both parameters reach zero (signifying the complete cessation of oscillatory activity).

**Fig 8:**
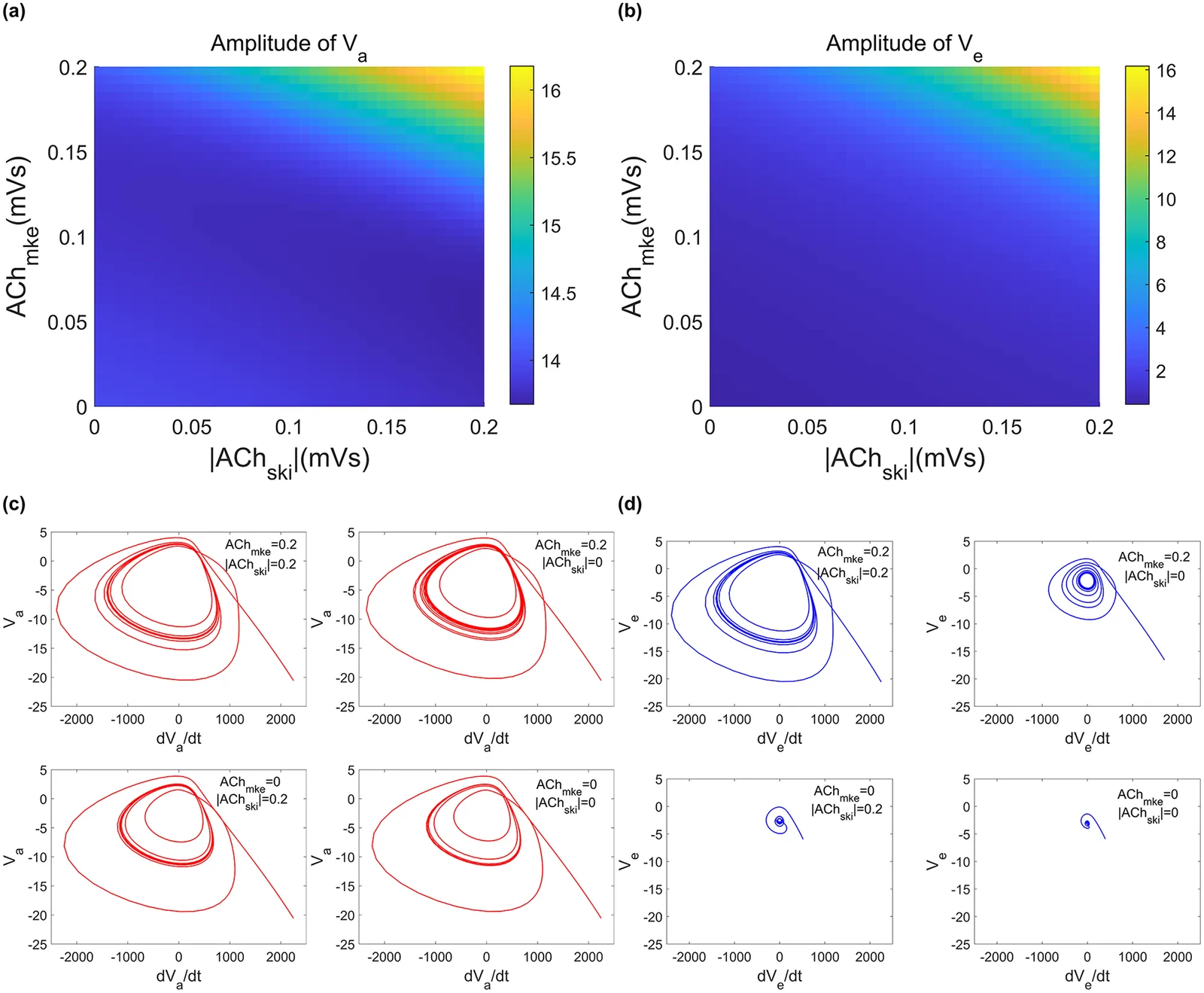
The projection diagrams of the amplitudes of $V_{a}$ (a) and $V_{e}$ (b) varying with ${ACh}_{mke}$ and ${|ACh}_{ski}|$. The phase planar graph of $V_{a}$ and its derivatives(c), the phase planar graph of $V_{e}$ and its derivatives(d) with respect to ${ACh}_{mke}$ and ${|ACh}_{ski}|$. From left to right, ${|ACh}_{ski}|$ decreases progressively (from 0.2 to 0), and from top to bottom, ${ACh}_{mke}$ decreases progressively (from 0.2 to 0), representing increasing severity of ACh deficiency in the associative circuit.

### 3.3 The impact of combined deficiencies in ACh and dopamine on cortical activity

In this subsection, we simulate dopamine deficiency in the motor and associative circuits by increasing the parameters $\left|A_{lg}\right|$ and $\left|A_{ms}\right|$, respectively. We decrease ${ACh}_{lke}$ and ${|ACh}_{gki}|$ to simulate ACh deficiency in the motor circuit, and decrease ${ACh}_{mke}$ and ${|ACh}_{ski}|$ to simulate ACh deficiency in the associative circuit, in order to systematically explore the effects of dual dopamine and ACh deficiency on cortical neural activity. Fig 9a shows the trend of the power ratio of $\phi_{a}$ with ${ACh}_{lke}$ and $\left|{ACh}_{gki}\right|$ under different $A_{lg}$. It can be seen that when ${|A}_{lg}|$ is relatively small, the power ratio of $\phi_{a}$ gradually decreases as the values of $A_{lke}$ and $\left|{ACh}_{gki}\right|$ decrease; When $\left|A_{lg}\right|$ is relatively large, due to severe dopamine deficiency, the power ratio of $\phi_{a}$ does not show significant changes as the absolute values of $A_{lke}$ and ${|ACh}_{gki}|$ decrease. This indicates that in the motor circuit, the deficiency of dopamine and ACh both leads to a decrease in the $\phi_{a}$ power ratio, but when one neurotransmitter is highly deficient, the effect of the other on the power ratio does not add up nonlinearly. Similarly, the power ratio of $\phi_{e}$ also shows the same trend with increasing $\left|A_{lg}\right|$ or decreasing values of ${ACh}_{lke}$ and ${|ACh}_{gki}|$ (Fig 9b). Furthermore, from the three-dimensional slice plot of $\phi_{a}$ as a function of ${|A}_{ms}|$, ${ACh}_{lke}$, and ${|ACh}_{gki}|$ (Fig 9c), it can be seen that compared to the decrease in the values of ${ACh}_{lke}$ and ${|ACh}_{gki}|$, the increase in ${|A}_{ms}|$ has a weaker contribution to the decrease in $\phi_{a}$ power ratio. In the variation of $\phi_{e}$, the increase of ${|A}_{ms}|$ significantly weakens its power ratio, while the changes in the values of ${ACh}_{lke}$ and ${|ACh}_{gki}|$ have little effect on its power ratio. Overall, the deficiency of dopamine and ACh can lead to a decrease in cortical neural activity, and the two have a synergistic effect, but not a simple linear additive relationship.

**Fig 9:**
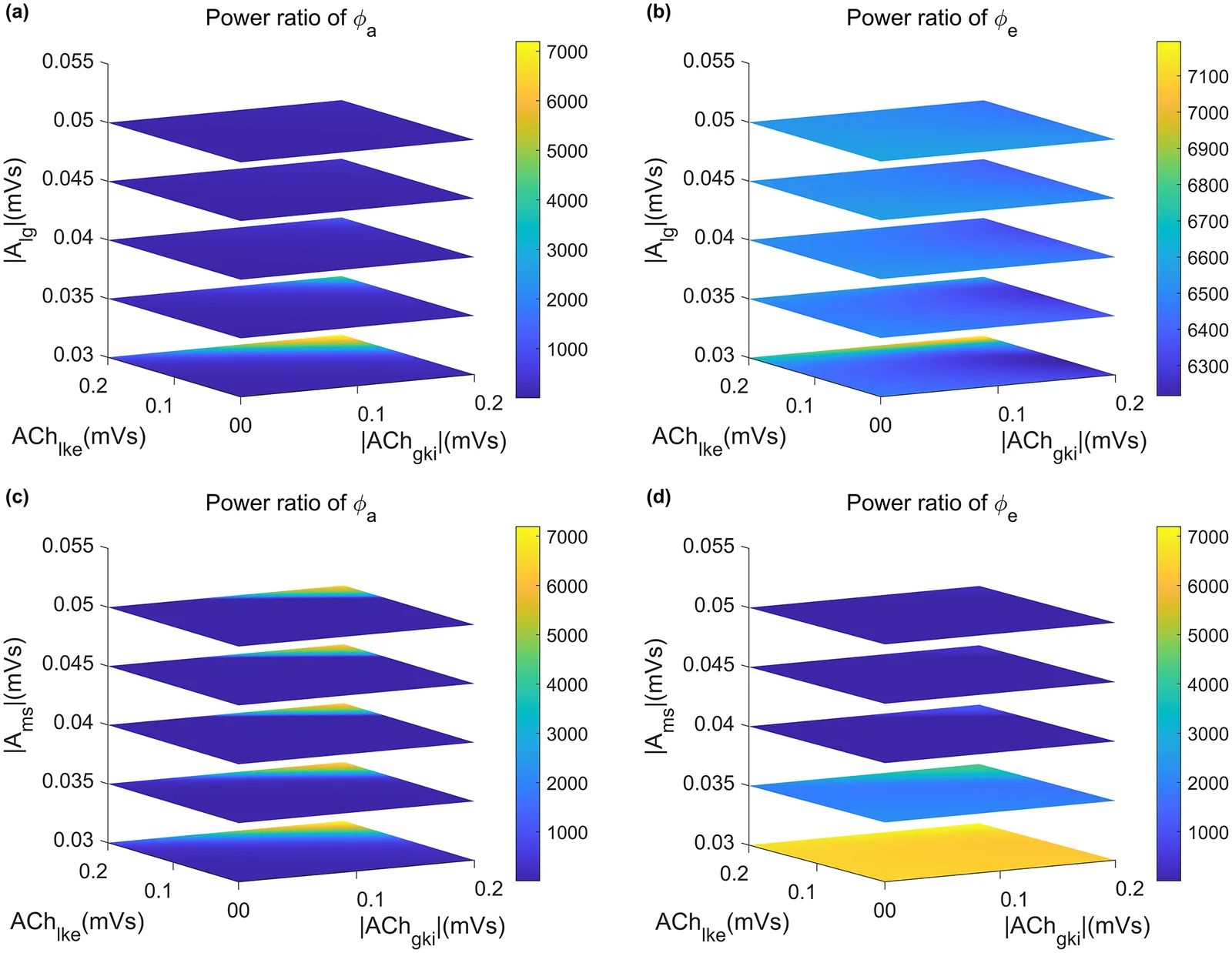
The slice maps of the power ratio of $\phi_{a}$ (a) and $\phi_{e}$ (b) with ${ACh}_{lke}$ and ${|ACh}_{gki}|$ under different $\left|A_{lg}\right|$. The slice maps of the power ratio of $\phi_{a}$ (c) and $\phi_{e}$ (d) with ${ACh}_{lke}$ and ${|ACh}_{gki}|$ under different ${|A}_{ms}|$.

In Fig 10, we simulate the combined effects of ACh deficiency in the associative circuit and dopamine deficiency in the two circuits on cortical activity. As illustrated in Fig 10a, when ${|A}_{lg}|$ is relatively small, a reduction in the values of ${ACh}_{mke}$ and ${|ACh}_{ski}|$ results in a slight attenuation of $\phi_{a}$; however, when $\left|A_{lg}\right|$ is relatively large, this reduction has minimal impact on $\phi_{a}$. Fig 10b demonstrates that regardless of ${|A}_{lg}|$ magnitude, decreased ${ACh}_{mke}$ and ${|ACh}_{ski}|$ lead to a significant reduction in $\phi_{e}$. Under conditions of higher ${|A}_{ms}|$, a decrease in the values of ${ACh}_{mke}$ and ${|ACh}_{ski}|$ causes a modest decline in the $\phi_{a}$ power ratio (Fig 10c); in contrast, under lower $A_{ms}$, this change exerts almost no effect on $\phi_{a}$, suggesting that dopamine deficiency in the associative circuit has a more pronounced influence on neural activity in the supplementary motor area than ACh deficiency. Moreover, the results in Fig 10d indicate that as $\left|A_{ms}\right|$ increases, reductions in the values of ${ACh}_{mke}$ and ${|ACh}_{ski}|$ both contribute to a marked decrease in the $\phi_{e}$ power ratio, although the interaction does not follow a simple linear superposition. Additionally, compared with the findings in Fig 9, it is evident that under normal dopamine levels, ACh deficiency in the motor circuit markedly suppresses prefrontal cortical neural activity, whereas this inhibitory effect becomes less apparent under severe dopamine deficiency. In contrast, the absence of ACh in the associative circuit consistently leads to a significant reduction in prefrontal cortical neural activity across all dopamine levels.

**Fig 10:**
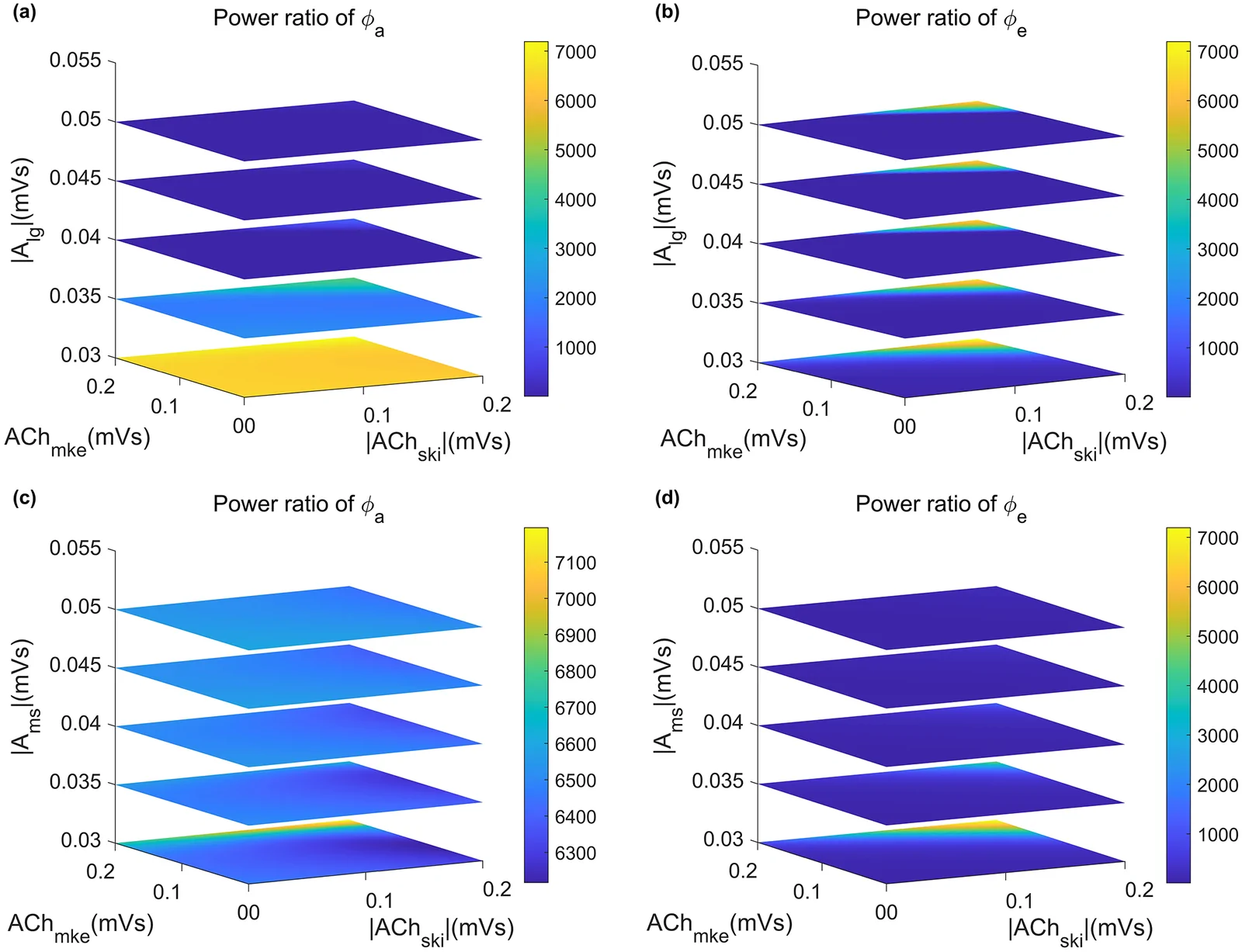
The slice maps of the power ratio of $\phi_{a}$ (a) and $\phi_{e}$ (b) with ${ACh}_{mke}$ and $\left|{ACh}_{ski}\right|$ under different ${|A}_{lg}|$. The slice maps of the power ratio of $\phi_{a}$ (c) and $\phi_{e}$ (d) with ${ACh}_{mke}$ and $\left|{ACh}_{ski}\right|$ under different ${|A}_{ms}|$.

### 3.4 Robustness analysis of the main findings

To evaluate whether the observed spectral slowing under ACh deficiency is robust to parameter uncertainty, we perform a local robustness analysis by perturbing three key parameters ($A_{ee}$, $A_{em}$, and $\theta_{e}$). For each perturbed condition, we systematically vary the four cholinergic parameters (${ACh}_{lke}$, ${ACh}_{gki}$, ${ACh}_{mke}$, and ${ACh}_{ski}$) and computed the power ratio of $\phi_{a}$ and $\phi_{e}$.

Fig 11 shows the variation of the power ratio with the four cholinergic parameters under different $A_{ee}$ values (nominal: 1.6). The upper panel presents results for $\phi_{a}$, and the lower panel for $\phi_{e}$. Across all $A_{ee}$ values, the power ratio consistently decreases as the cholinergic parameters are progressively reduced. Although the absolute power ratio values shift with changes in $A_{ee}$, the monotonic decreasing trend under ACh deficiency remains unchanged, confirming that the spectral slowing effect is robust to variations in cortical excitatory strength.

**Fig 11:**
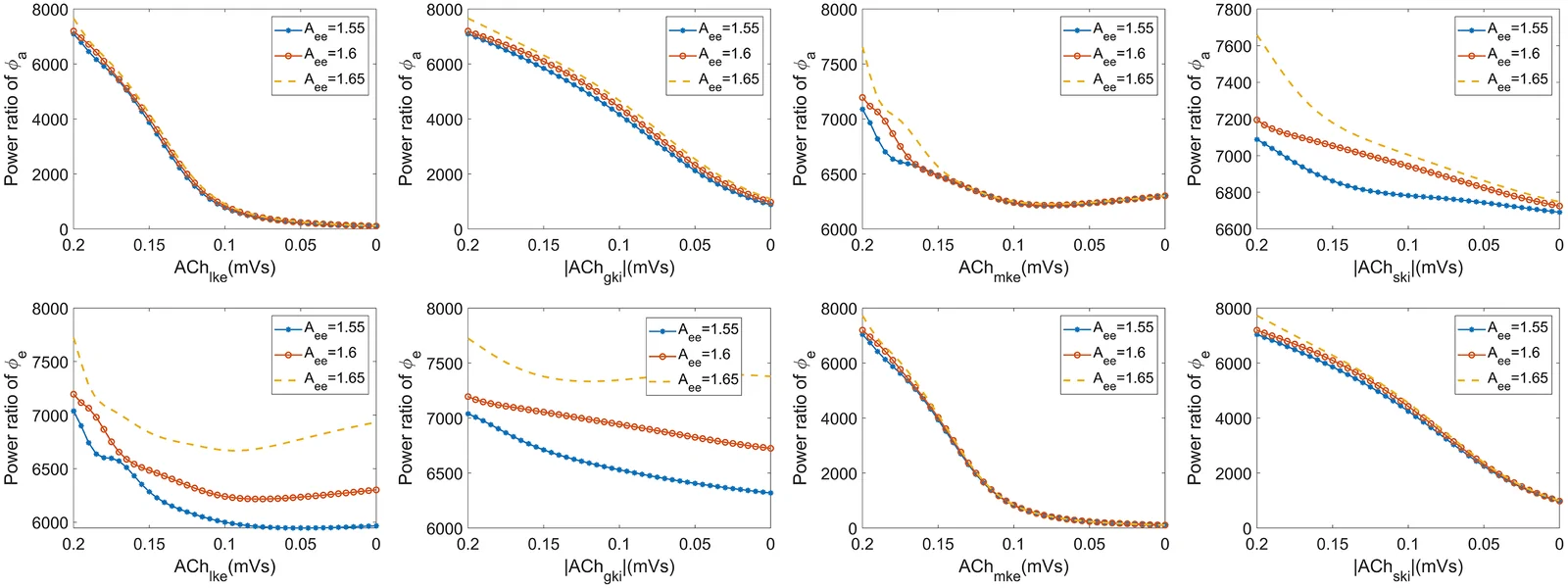
The variation of the power ratio of $\phi_{a}$ (upper panel) and $\phi_{e}$ (lower panel) with acetylcholine-related parameters (${ACh}_{lke}$, ${ACh}_{gki}$, ${ACh}_{mke}$, and ${ACh}_{ski}$) under different $A_{ee}$.

Fig 12 presents the same analysis under different $A_{em}$ values (nominal: 0.8). For both $\phi_{a}$ and $\phi_{e}$, the power ratio shows a consistent decline with progressive ACh deficiency across all perturbed $A_{em}$ values. The pattern of decrease is preserved despite modest variations in the absolute power ratio, indicating that the thalamocortical excitatory strength does not qualitatively affect our main conclusion.

**Fig 12:**
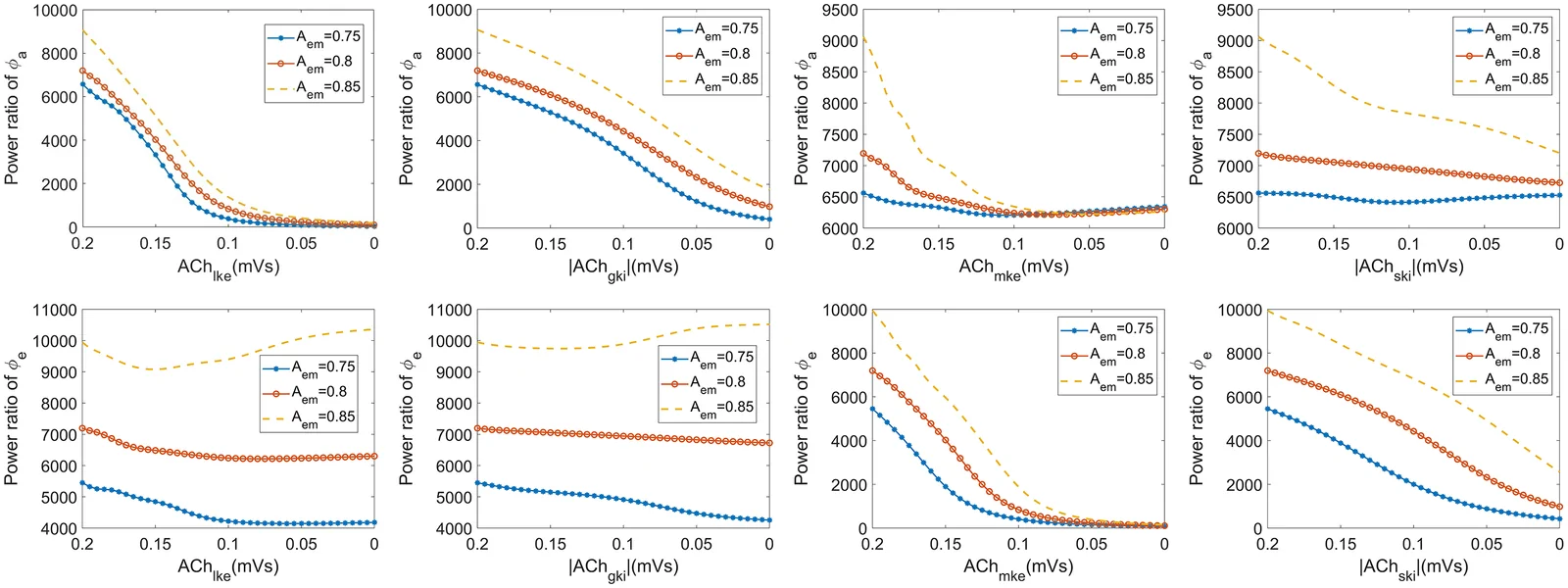
The variation of the power ratio of $\phi_{a}$ (upper panel) and $\phi_{e}$ (lower panel) with acetylcholine-related parameters (${ACh}_{lke}$, ${ACh}_{gki}$, ${ACh}_{mke}$, and ${ACh}_{ski}$) under different $A_{em}$.

Fig 13 displays the results under different $\theta_{e}$ values (nominal: 14 mV). Varying the cortical firing threshold does not alter the qualitative finding: the power ratio consistently decreases as cholinergic parameters are reduced, for both $\phi_{a}$ and $\phi_{e}$.

**Fig 13:**
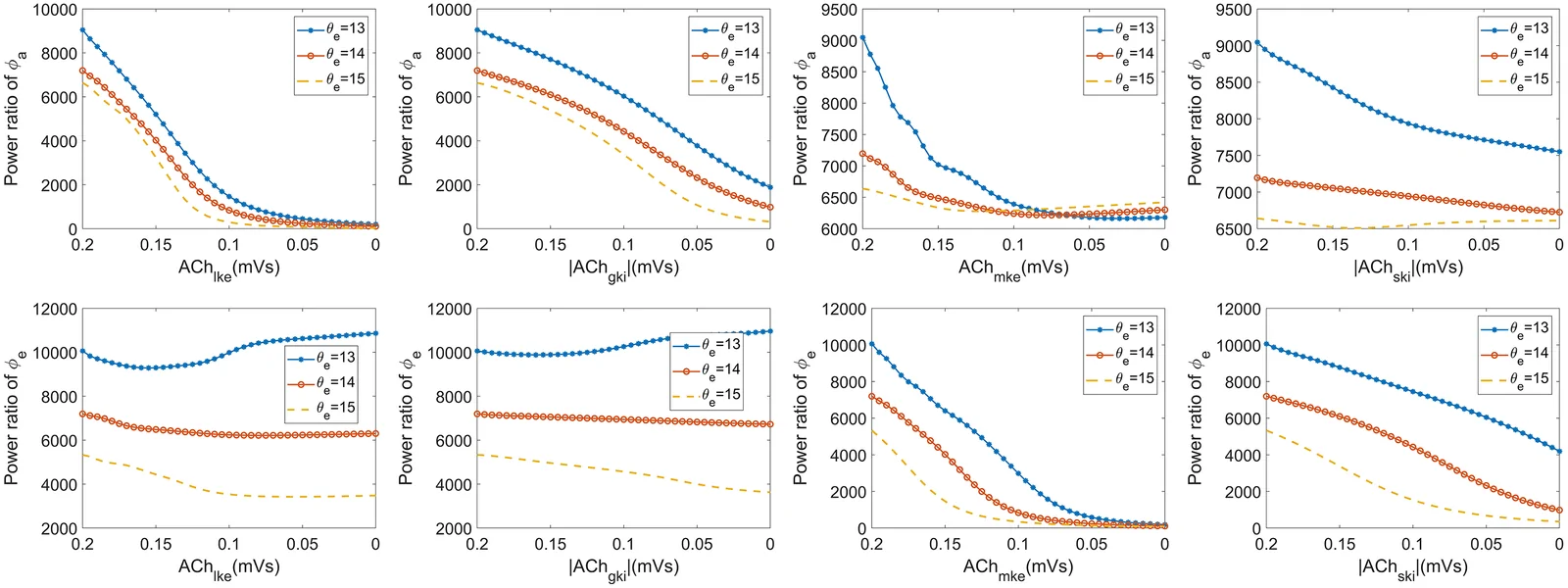
The variation of the power ratio of $\phi_{a}$ (upper panel) and $\phi_{e}$ (lower panel) with acetylcholine-related parameters (${ACh}_{lke}$, ${ACh}_{gki}$, ${ACh}_{mke}$, and ${ACh}_{ski}$) under different $\theta_{e}$.

In summary, across all perturbed conditions, the direction of the effect—a monotonic decrease in the high/low frequency power ratio under progressive ACh deficiency—remained unchanged. While the absolute magnitudes of the power ratio varied modestly with parameter perturbations, the qualitative conclusion that ACh deficiency induces spectral slowing is robust to the variations in these core parameters. This confirms that our main findings are not artifacts of a single parameter set and are qualitatively reliable despite the inherent variability in biological parameters.

## 4. Conclusion and discussion

Given that the degeneration of cholinergic neurons in PPN represents a key pathological feature in PDD, complementary to dopaminergic dysfunction, this study develops an enhanced computational model of the basal ganglia-thalamus-cortex circuit incorporating the PPN. By systematically modulating critical synaptic connections, we investigate how ACh deficiency affects neural activity in the supplementary motor area and prefrontal cortex and how cholinergic and dopaminergic deficits may jointly influence cortical network dynamics.

Firstly, ACh deficiency in the motor circuit is computationally modeled by decreasing the parameter ${ACh}_{lke}$ and ${|ACh}_{gki}|$. Results demonstrate that as the values of ${ACh}_{lke}$ and ${|ACh}_{gki}|$ decreased, the power ratios of $\phi_{a}$ and $\phi_{e}$ both decline. Notably, the reduction in $\phi_{a}$ is markedly pronounced (dropping from approximately 7000 to nearly zero), whereas the decline in $\phi_{e}$ is comparatively modest. This decline trend of spectral power aligns with the characteristic rhythm slowing observed in EEG recordings from patients with PD [4–8], suggesting that ACh depletion in the motor circuit is associated not only with reduced neural activity in the supplement motor area but also with attenuated neural activity in the prefrontal cortex. Biologically, in the motor circuit, the pathways from PPN to VL (${ACh}_{lke}$) and VAMC (${ACh}_{gki}$) synergistically excite VL. Reducing both weakens thalamic drive, causing near-complete collapse of supplement motor area oscillations. The prefrontal cortex is indirectly affected via putamen-SNr connections, accounting for its modest decline. Under severe deficiency, the VL becomes hypoactive and fails to sustain cortical rhythmicity.

Secondly, ACh deficiency is simulated within the associative circuit by reducing ${ACh}_{mke}$ and ${|ACh}_{ski}|$. Under this condition, the power ratio of $\phi_{a}$ exhibits a downward trend with decreasing the values of ${ACh}_{mke}$ and ${|ACh}_{ski}|$, consistent with prior EEG-based observations in PD [7,8]. Concurrently, the power ratio of $\phi_{e}$ declines more substantially, reflecting the heightened vulnerability of associative circuit in PD-associated dementia. Furthermore, dynamical analysis on time-series reveals that the decrease in the power ratio is accompanied by a progressive weakening of oscillatory activity and the firing pattern transits from limit cycle to point attractor. This suggests that the decrease of power ratio may stem from weakened oscillatory activity. In the associative circuit, reducing ${|ACh}_{ski}|$ and ${ACh}_{mke}$ weaken VAMC disinhibition, increasing inhibition of the prefrontal cortex. This greater sensitivity explains why prefrontal dysfunction is more prominent in PDD. Dynamically, ACh deficiency reduces system excitability below the threshold for sustained oscillations, effectively quenching rhythmic activity.

Finally, we investigate the effect of the absence of dual neurotransmitters on cortical activity. The simulated results imply that while both ACh and dopamine deficits independently suppress $\phi_{a}$ or $\phi_{e}$ power, their interaction is non-additive: severe impairment in one system occludes the functional impact of perturbations in the other.

This study presents a computational investigation of ACh deficiency in PDD. The simulation outputs qualitatively align with experimental observations and offer a candidate mechanism for further investigation. Nevertheless, several aspects of the current model could be refined. First, the integration of striatal cholinergic neuron dysfunction should be addressed. Second, a systematic exploration of the parameter space is currently lacking. Due to the model’s high dimensionality and the presence of transcendental functions in the firing rate expression, global sensitivity or bifurcation analysis is computationally demanding and analytically challenging. In this study, we rely on numerical simulations using parameter values adopted from established literature, which are physiologically grounded. Therefore, we believe the current parameter settings are biologically plausible. However, a thorough high-dimensional parameter exploration remains an important direction for future research, which may potentially be tackled using surrogate modeling or dimensionality reduction techniques to overcome both computational and analytical challenges. Finally, the current model omits the GPe and STN, both of which are known to contribute to abnormal oscillations of PD, particularly in the beta band. Incorporating these structures in future model extensions would enable a more complete understanding of how cholinergic and dopaminergic deficits interact across the full basal ganglia circuit. Addressing these improvements would contribute to more complete understanding of the relationship between aberrant ACh signaling and PDD.

## Supporting information

S1 File Supporting information for this study, including MATLAB code for simulations and figure generation and the associated figure files. (ZIP)
